# Dynamics of digestive proteolytic system during blood feeding of the hard tick *Ixodes ricinus*

**DOI:** 10.1186/1756-3305-3-119

**Published:** 2010-12-14

**Authors:** Zdeněk Franta, Helena Frantová, Jitka Konvičková, Martin Horn, Daniel Sojka, Michael Mareš, Petr Kopáček

**Affiliations:** 1 Institute of Parasitology, Biology Centre, Academy of Sciences of the Czech Republic, Branišovská 31, České Budějovice, CZ-370 05, Czech Republic; 2 Faculty of Science, University of South Bohemia, Branišovská 31, České Budějovice, CZ-370 05, Czech Republic; 3 Institute of Organic Chemistry and Biochemistry, Academy of Sciences of the Czech Republic, Flemingovo nám. 2, Prague 6, CZ-166 10, Czech Republic

## Abstract

**Background:**

Ticks are vectors of a wide variety of pathogens causing severe diseases in humans and domestic animals. Intestinal digestion of the host blood is an essential process of tick physiology and also a limiting factor for pathogen transmission since the tick gut represents the primary site for pathogen infection and proliferation. Using the model tick *Ixodes ricinus*, the European Lyme disease vector, we have previously demonstrated by genetic and biochemical analyses that host blood is degraded in the tick gut by a network of acidic peptidases of the aspartic and cysteine classes.

**Results:**

This study reveals the digestive machinery of the *I. ricinus *during the course of blood-feeding on the host. The dynamic profiling of concentrations, activities and mRNA expressions of the major digestive enzymes demonstrates that the *de novo *synthesis of peptidases triggers the dramatic increase of the hemoglobinolytic activity along the feeding period. Overall hemoglobinolysis, as well as the activity of digestive peptidases are negligible at the early stage of feeding, but increase dramatically towards the end of the slow feeding period, reaching maxima in fully fed ticks. This finding contradicts the established opinion that blood digestion is reduced at the end of engorgement. Furthermore, we show that the digestive proteolysis is localized intracellularly throughout the whole duration of feeding.

**Conclusions:**

Results suggest that the egressing proteolytic system in the early stage of feeding and digestion is a potential target for efficient impairment, most likely by blocking its components via antibodies present in the host blood. Therefore, digestive enzymes are promising candidates for development of novel 'anti-tick' vaccines capable of tick control and even transmission of tick-borne pathogens.

## Background

Ticks are blood-sucking ectoparasites that may do harm to their host by severe blood loss, but the main danger of ticks remain their capability to transmit a wide variety of pathogens comprising viruses, bacteria and protozoa [1], causing diseases in humans and animals [2]. The miscellaneous vectorial capacity of ixodid ticks is due to their long-term co-evolution with the pathogens that they transmit, extended lifespan (up to years), the long-lasting blood feeding of all parasitic stages on different hosts and other aspects of tick biology [3]. Moreover, the transmission of pathogens is facilitated by the modulation of host haemostatic, inflammatory and immune responses, mediated by the inexhaustible pharmacology of the molecules present in tick saliva [4]. The castor bean tick, *Ixodes ricinus *is the most important arthropod disease vector in Europe, notorious for transmitting the tick-borne encephalitis virus and the Lyme disease spirochetes of the genus *Borrelia *[5], supporting the importance of genome sequencing of the closely related American deer tick, *Ixodes scapularis *[6]. During blood feeding, *Borrelia sp*. spirochetes multiply in the tick gut lumen and change the expression pattern of their outer surface proteins, allowing them to exit the gut and migrate to salivary glands, which they enter to ultimately infect the mammalian host [7]. These data suggest that transmission of *Borrelia sp*. and other tick borne pathogens can be potentially controlled via an efficient impairment of blood feeding and digestion. In our previous work, we have demonstrated in semi-engorged *I. ricinus *females that intestinal digestion is based on an evolutionarily conserved multienzyme complex of cysteine and aspartic peptidases [8], orthologous to those characterized in other parasites, including platyhelminthes [9,10] and nematodes [11]. We have also mapped the hemoglobinolytic cascade revealing the successive involvement of individual enzymes [12]. Hemoglobin digestion in the *I. ricinus *gut is initiated by generation of large fragments by aspartic peptidase of the cathepsin D-type (IrCD), supported by the cysteine class peptidase of the papain-type cathepsin L (IrCL) and legumain-type asparaginyl endopeptidase (IrAE). The cleavage of large hemoglobin fragments is further mediated by another papain-type cysteine endopeptidase cathepsin B (IrCB) and completed by the amino- and carboxydipeptidase activity of cathepsin C (IrCC) and IrCB, respectively [12]. Since the genetic screening [8] as well as the proteomic characterization of the *I. ricinus *digestive pathway [12] was performed for a single-time point towards the end of the slow feeding period (the sixth day post-attachment), several principle questions concerning the roles of the individual peptidases during tick feeding remain to be answered: (i) what is the dynamic course of overall hemoglobinolysis and the activity of component peptidases in relation to the feeding phases? (ii) is the hemoglobinolytic pathway (network) preserved during the entire blood feeding? (iii) is the digestion regulated at the transcriptional, translational or pro-enzyme activation level? (iv) is the site of action of hemoglobinolytic enzymes spatially restricted to the gut digestive cells throughout the whole feeding period? In order to shed more light into the process of blood uptake and digestion during tick feeding on the host, we performed complex dynamic analysis of the gene expression, activity and molar concentrations of the major digestive peptidases. In addition, indirect immunofluorescent localization of the major peptidase of the hemoglobinolytic pathway, IrCB, made it possible to follow the development of the digestive machinery in the gut cells from unfed to fully engorged females. This work provides a first, comprehensive insight into the dynamics of the digestive apparatus in an important tick model indicating the potential of blood feeding impairment as an efficient tick control point.

## Results

### Morphological changes in the *Ixodes ricinus *gut during feeding correlate with overall hemoglobinolytic capacity

**Figure 1:**
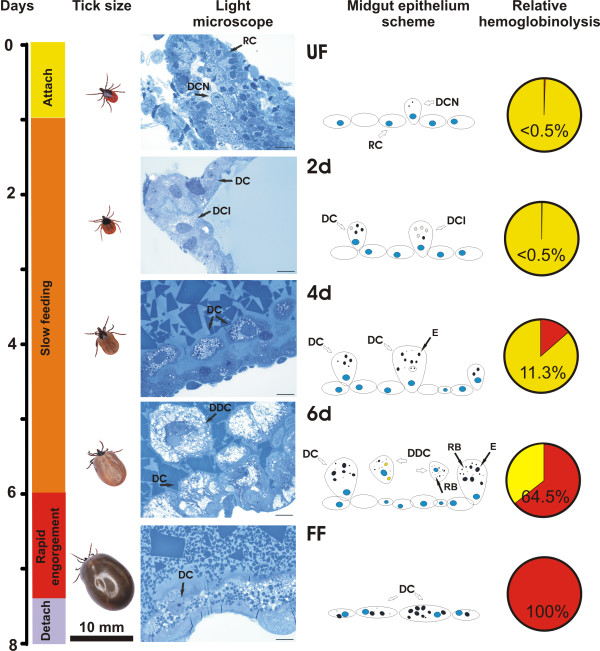
**Overview of the feeding phases, midgut morphology and overall hemoglobinolysis in the gut of a *Ixodes ricinus *female during feeding on the host**. An adult *Ixodes ricinus *female feeds for about 7 to 8 days. The slow feeding period starts one day post-attachment, during which the female ingests about one third of the total blood meal. The major portion of the host blood (about two thirds) is ingested by the mated female during the rapid engorgement phase, taking place during the last 24-48 hours before the engorged tick drops off the host. Light microscope panel: The semi-thin sections were stained with toulidine blue; scale bar = 20 μm. **UF **- unfed ticks; **2d**, **4d, 6d **- 2, 4, 6 days of feeding, respectively; **FF **- fully fed (engorged) ticks. Midgut epithelium scheme panel: **RC **- reserve cells (stem cells); **DCN **- digestive cells persisting from the nymphal stage; **DCI **- initial digestive cells (prodigest cells); **DC **- digestive cells; **DDC **- detached digestive cells; **RB **- residual bodies (hemosomes); **E **- endosomes; blue circles - cell nuclei; yellow circles - lipid vacuoles. Relative hemoglobinolysis panel: Quantification of relative hemoglobinolysis in gut tissue extracts was measured using the fluorescamine derivatization assay at pH 4.2 and normalized to one tick gut according to the method described in reference [12].

Feeding of adult *I. ricinus *females on guinea pigs takes 7 to 8 days under in-house laboratory conditions. The feeding phases as known for the ixodid ticks [3,13] are schematically depicted and aligned with the corresponding observed gut morphological changes and overall hemoglobinolytic activities (Figure 1). During attachment, narrow gut lumen of unfed ticks is surrounded by the midgut epithelium formed by undifferentiated reserve cells (also referred to as replacement cells [13] or stem cells [14]) and digestive cells preserved from the preceding nymphal stage. On the second day post attachment (p.a.), the midgut lumen slowly expands with the uptake of the first portion of host blood and the reserve cells start to grow and differentiate into the initial digestive cells, also named prodigest cells [15]. The hemoglobinolytic activity in gut tissue extracts (GEs) of unfed as well as 2-day-fed ticks is barely detectable. On the fourth day of feeding, the digestive cells further enlarge and display signs of hemoglobin uptake and digestion. The first residual bodies containing waste aggregated heme products, formed in organelles called hemosomes [16], as well as large endosomes and lipid vacuoles, start to appear at this time point (Figure 1). During this phase of slow feeding, it is already possible to observe the detachment of the first digestive cells from the midgut epithelium and the initial increase in overall hemoglobinolytic activity. The most dramatic changes in the midgut morphology is evident towards the end of the slow feeding period, about the six days p.a.. At this stage, the digestive cells are fully extended, filled with residual bodies, large endosomes and lipid inclusions. The detached digestive cells are liberated into the lumen without any sign of lysis and most likely removed by defecation. The hemoglobinolytic activity in the gut tissue extract in semi-engorged ticks (six days p.a.) reaches about 65% of the maximum. The rapid-engorgement phase occurs during the last 24-48 hours prior to detachment. It has been referred to as the 'big-sip' [3] because mated females imbibe about two-thirds of the entire blood-meal within this phase. The appearance of the midgut in fully fed females differs from that observed during the slow feeding period. The midgut epithelium is overlaid with flattened sessile digestive cells that remain associated with the epithelium; reserve or initial digestive cells are not detected anymore. Noticeably, the overall hemoglobinolytic activity reaches its maximum in gut extracts of fully engorged ticks that just dropped off the host.

### The amount of component digestive peptidases markedly increases towards the end of slow feeding

Two approaches were employed to quantify the component digestive peptidases in GEs: (i) the molar content of peptidases was determined by active-site titration with selective irreversible inhibitors; (ii) the enzymatic activity of peptidases was measured in a kinetic assay with specific fluorogenic substrates.

**Figure 2:**
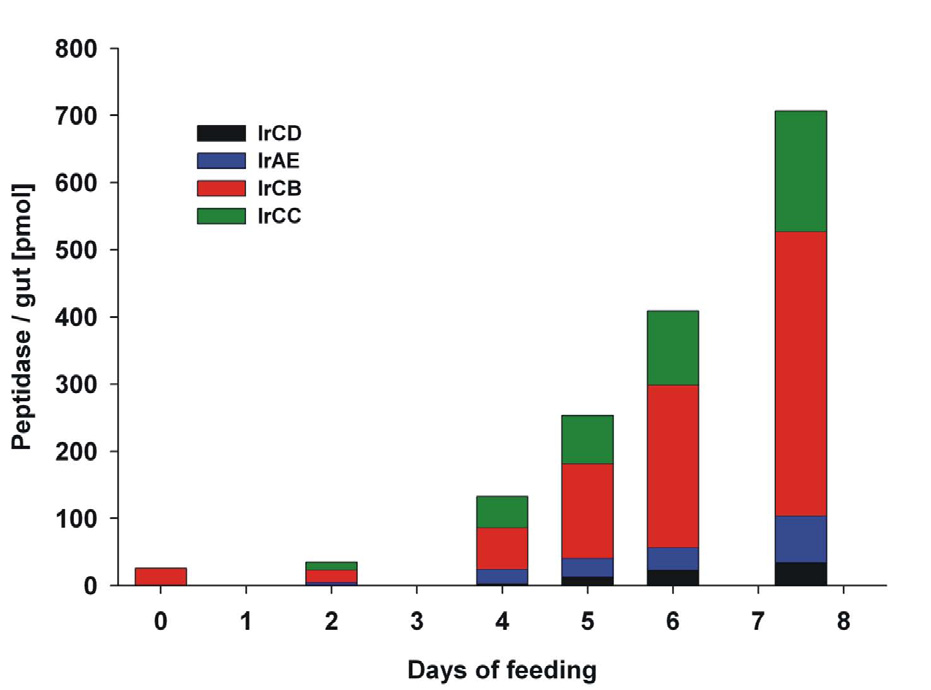
**Active-site titration of digestive peptidases in the *Ixodes ricinus *gut tissue extracts during feeding on the host**. The groups of 15-20 females were forcibly removed and collected from four guinea pigs at the indicated feeding time points. The guts from individual ticks were subsequently dissected and the gut tissue extracts were prepared from the pool of longitudinally cut midgut halves. The absolute molarities of the individual peptidases in gut homogenates were determined by stoichiometric titration with the appropriate active-site inhibitors (Table 1). Bars represent the average value from a triplicate measurement. Standard deviations were ≤10% of the average value and are not shown. The molarity of cathepsin L (IrCL) was not determined because an appropriate inhibitor was lacking. For details, see Material and Methods section.

The active-site titration was performed for all enzymes except for IrCL, due to the lack of an appropriate irreversible inhibitor as a titrant (Table 1). The concentrations of digestive peptidases proportionally increase in time and reach their maximum levels in fully fed ticks (Figure 2). IrCB is the dominant enzyme throughout the whole feeding period and it is the only enzyme which can be detected in the GE of unfed ticks. The second most abundant enzyme is the dipeptidyl peptidase IrCC. The amounts of IrCB and IrCC exceed the concentrations of the other peptidases in the network by more than one order of magnitude.

**Table 1:** Assay conditions for the fluorimetric measurements of digestive peptidases activities and stoichiometric active-site titration

Enzyme	pH	Substrate (final concentration)	Shielding inhibitor (final concentration)	Excitation/Emission [nm]	Active-site titration inhibitor
**IrCB**	5.5	Z-Arg-Arg-AMC^a^ (5 μM)	-	360/465	CA-074^c^

**IrCL**	4.0	Z- Phe-Arg-AMC^a^ (5 μM)	CA-074^c ^(2.5 μM)	360/465	n.d.^e^

**IrCC**	5.5	Gly-Arg-AMC^a^ (40 μM)	-	360/465	Ala-Hph-VS-Ph^f^

**IrAE**	5.5	Z-Ala-Ala-Asn-AMC^a^ (10 μM)	CA-074^c ^(2.5 μM)	360/465	Aza-N-11a^g^

**IrCD**	4.0	Abz-Lys-Pro-Ala-Glu-Phe-Nph-Ala-Leu^b^ (32 μM)	E-64^d ^(5 μM)	330/425	pepstatin^h^

^a ^The fluorogenic AMC-substrates (AMC, 7-amino-4-methylcoumarin) were from Bachem

^b ^Fluorescence resonance energy transfer (FRET) substrate [38]

^c ^Epoxysuccinyl-based inhibitor of cathepsin B [39]

^d ^Epoxysuccinyl-based inhibitor of papain-type cysteine peptidases (cathepsins C, B and L) [34]

^e ^n.d. - not determined due to a lack of an appropriate titrant

^f ^Vinyl sulfone-based inhibitor of cathepsin C [40]

^g ^Aza-peptide Michael acceptor-based inhibitor of asparaginyl endopeptidases [41]

^h ^Statine-based inhibitor of aspartic peptidases [35].

**Figure 3:**
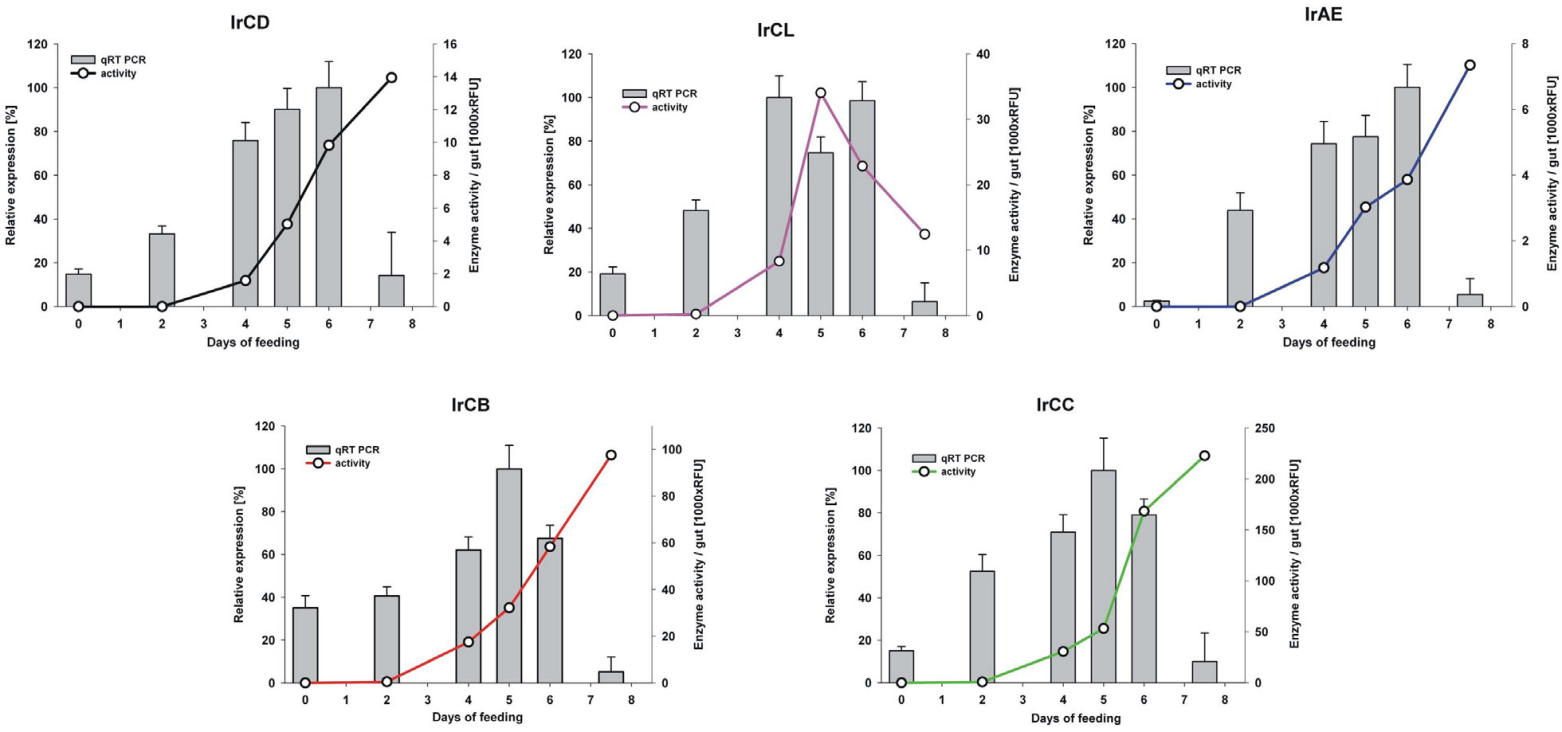
**Dynamic profiles of mRNA expression and enzyme activities of individual peptidases during the feeding of *Ixodes ricinus *females**. For each time point, the guts were dissected from 15-20 females removed from four guinea pigs. Half of the guts were processed either for total RNA isolation or gut tissue extraction as described above. Gene expression profiles of the indicated enzymes were determined by qRT-PCR, using elongation factor 1 as a reference gene. The expressions were related to the maximum mRNA level set as 100%. Columns correspond to the average value of triplicate technical determinations, while error bars indicate the corresponding standard deviations. The enzyme activity curves of indicated peptidases were determined using specific fluorogenic substrates and shielding inhibitors to mask possible interference from other peptidases (see Table 1). Enzymatic activities in the homogenates were normalized per one gut tissue. The circles display the average activities obtained from triplicate measurements. The standard deviations were ≤5% of the average value and are not shown.

The activity measurements of individual enzymes in the GEs were performed at their pH optima with specific fluorogenic substrates, preventing the confounding hydrolysis activities of other peptidases with the aid of shielding inhibitors (Table 1). In accordance with the active-site titration results, IrCB was the only enzyme giving measurable activities in the gut extracts from unfed ticks. As shown in the Figure 3 the activities of IrCD, IrAE, IrCB and IrCC, respectively, begin to increase after the second day of feeding, followed by an exponential increase between days 4 and 6 and finally reaching their maxima in fully fed ticks. The only enzymatic activity that proved to be an exception to this trend was that of IrCL, which peaks by the fifth day of feeding and then steeply declines in fully fed ticks (Figure 3).

### Digestive peptidases are regulated at the transcriptional level

Determination of molar concentrations of individual digestive peptidases as well as their activity profiles during blood feeding prompted further studies to assess whether de-novo protein synthesis and/or activation of existing pro-enzymes is the mode of action underlying their increase. The determination of digestive peptidase mRNA levels by semi-quantitative real-time PCR (qRT-PCR) analysis demonstrated that the dynamic profiles of mRNA expression clearly precede the activities of their respective peptidases (Figure 3; bar graphs) implying that the major components of the digestive system are transcriptionally regulated. The mRNA levels of IrCB and IrCC reach their maxima already five days p.a., IrCD and IrAE mRNA levels peak six days p.a. and IrCL mRNA displays two expression maxima the fourth and the sixth day p.a.. For all enzymes, the mRNA expression drops dramatically in fully fed ticks, indicating that the further continual digestive phase in engorged ticks following their detachment from the host relies mainly on the enzymes synthesized during the slow feeding period.

### The digestive apparatus is localized intracellularly during the whole feeding period

**Figure 4:**
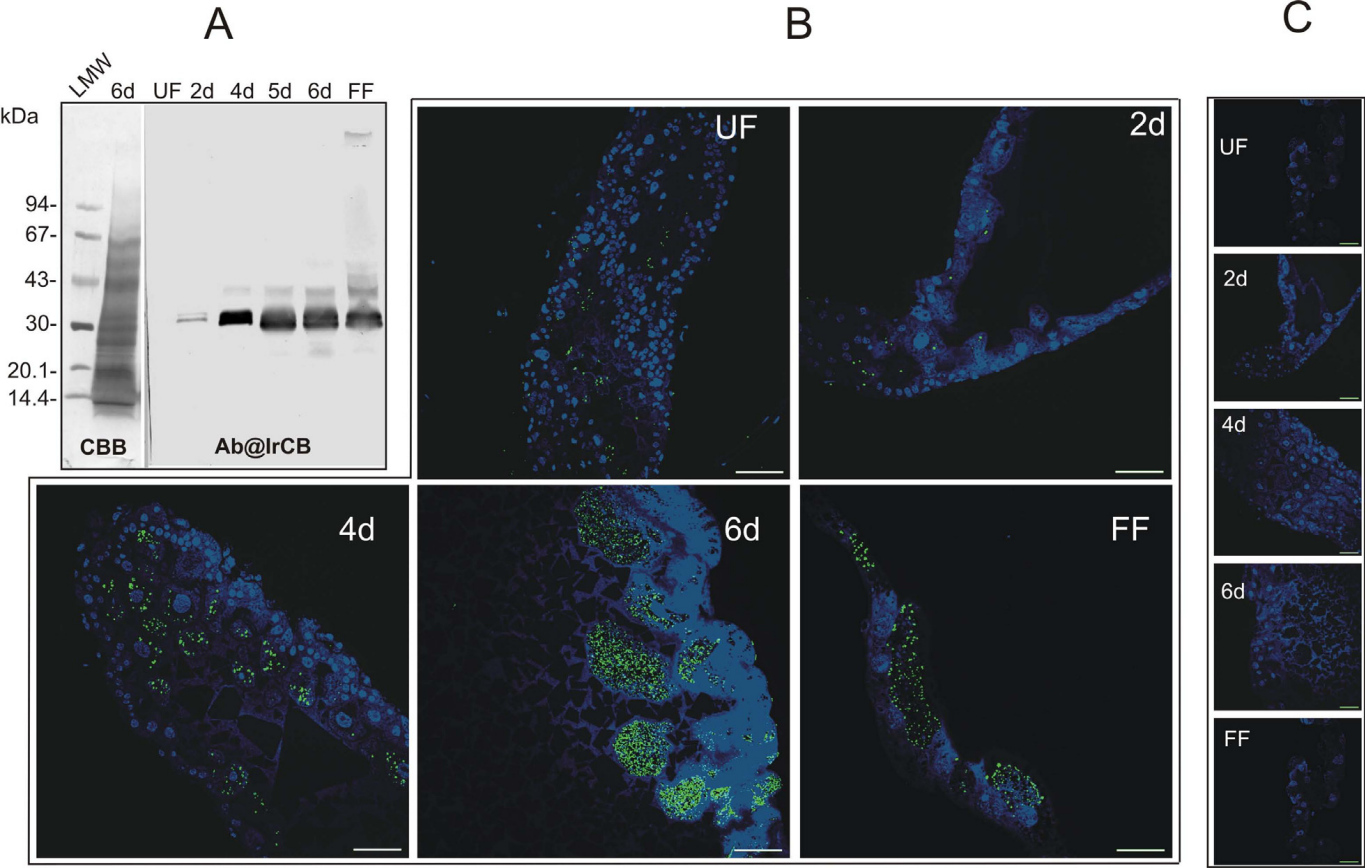
**Immunolocalization of cathepsin B (IrCB) in the gut of an *Ixodes ricinus *female during feeding**. Panel A: Western blot analysis of authentic IrCB in gut homogenates electro-transferred onto a PVDF membrane. LMW - protein markers; UF - unfed ticks; 2 d - 2 days fed ticks (0.2 gut/lane); 4 d, 5 d, 6 d - 4, 5, 6 days fed ticks, respectively (0.1 gut/lane); FF - fully fed ticks (0.08 gut/lane). CBB - Coomassie stained membrane; Ab@IrCB - rabbit antibody against recombinant IrCB (Ra×IrCB_Ig_, dilution 1:100). Secondary antibody - Swine anti-rabbit-peroxidase conjugate (1:1000). Panel B: Immunolocalization of IrCB in the gut of *I. ricinus *on semi-thin sections using affinity purified rabbit antibody Ra×IrCB_ACP _(30 μg/ml). Secondary antibody - Goat anti-rabbit with conjugated Alexa Fluor^® ^488 (1:200). Merged images with DAPI nuclei staining; scale bars = 20 μm. Panel C: Control images without primary antibodies.

To demonstrate whether the hemoglobinolytic machinery remains intracellular during the whole blood feeding, immunolocalization of IrCB, the most abundant peptidase of the network, was performed. The separation of gut tissue extracts by SDS-PAGE followed by immunoblot analysis using the antibody prepared against the recombinant IrCB labeled a faint band of about 38 kDa and an intense double band of 33 and 31 kDa (Figure 4A). The former band likely corresponds to the IrCB pro-enzyme having a theoretical mass (without glycosylation) of 35,725 Da [8], whereas the double band may correspond to the intermediate and fully activated form of IrCB, as has been previously described for schistosmal cathepsin B [17] or to a possible presence of another closely related isoform of IrCB that cross-reacts with the antibody [12]. Activated IrCB protein starts to appear at the second day of feeding and becomes quite intense from the fourth day on to the fully fed stage (Figure 4A). This result is consistent with the quantitative results obtained by active-site titration and activity measurements.

In order to minimize the background fluorescence of the gut tissues and to obtain a clear immunostaining signal, affinity purified anti-IrCB antibodies from the rabbit serum were used. Immunolocalization of IrCB in tick guts corresponding to specified feeding periods clearly demonstrates that IrCB gradually accumulates and remains restricted within the digestive cells during the entire duration of the tick feeding on the host (Figure 4B). Traces of IrCB were observed already in the gut sections of unfed ticks, possibly as a remnant of the enzyme from the preceding nymphal stage. The increase of IrCB in the digestive cells becomes clearly evident by the fourth day of feeding. Towards the end of the slow feeding period (day six p.a.), most of the digestive cells are fully loaded with the enzyme and detached from the epithelium. No evidence of disintegration of the digestive cells and release of the free enzyme into the lumen was observed. This finding was further corroborated by the control measurement of activity of digestive enzymes in the gut lumen, which did not exceed 10% of the activities determined in GE (data not shown). In fully fed ticks, the last generation of digestive cells containing the IrCB remains associated within the midgut epithelium. Although the intensity of the IrCB signal within the sessile digestive cells in fully fed ticks seems to be lower compared to the day six p.a. (Figure 4B, panels FF vs. 6d), the remarkable enlargement of the gut tissue epithelium apparently accounts for the apparent maximal hemoglobinolytic capacity of the whole gut tissue extract in fully fed ticks.

## Discussion

During previous studies we have described the major constituents of the digestive machinery of the hard tick *I. ricinus *and deciphered how they are organized into a hemoglobinolytic pathway [8,12]. Herein, we have focused on the dynamic profiling of the components of the tick digestive apparatus during the course of tick feeding. This information is particularly relevant since the feeding phase is decisive for pathogen transmission. We have provided a comprehensive insight into the gene transcription, activity and molar concentration of five digestive enzymes during blood feeding, as well as the localization of the IrCB, as the most abundant peptidase of the pathway. Placing these molecular data into the context of previous ultrastructural and histochemical studies on tick gut development performed in 1970's and 80's [3,13,18] improves significantly our understanding of the entire process of intracellular blood digestion.

Morphological changes of the midgut epithelium observed during blood feeding of *I. ricinus *by light microscopy (Figure 1) basically correspond to the numerous previous reports published on gut morphology in other ixodid tick species [14,15,19,20]. In these studies, a variety of cells within the gut epithelium have been described and named according to their structure and/or putative functions, resulting in a rather disperse nomenclature in different tick species. Based on our observations (Figure 1), we tend to support the hypothesis of some authors [13,15] that various cell types represent digestive cells at different stages of development, reflecting their growth, differentiation and gradually changing function in endocytosis, lysosomal digestion, secretion and handling of waste products via hemosome formation [16]. This study also supports the recognition of distinct digestive phases previously based on the structural changes of the tick gut [18]. The initial continual digestive phase takes place during the slow feeding period starting one to two days p.a. to the host and is characterized by the gradual maturation and detachment of digestive cells from the gut epithelium [13]. During the rapid engorgement phase, when the mated female ingests about two thirds of the total blood meal, no more reserve cells or initial digestive cells are observed and the mature digestive cells remain associated with the epithelium.

We have previously characterized the hemoglobinolytic pathway in semi-engorged *I. ricinus *females that were fed for six days, comprising of a cascade of enzymatic activities of aspartic peptidase IrCD, cysteine peptidase IrCL, asparaginyl endopeptidase IrAE, the major endo/exopeptidase IrCB and dipeptidyl peptidase IrCC [12]. The determination of the molar concentrations as well as activity measurements of the component peptidases presented in this work support that the hemoglobinolytic pathway described for the sixth day p.a. is preserved throughout the entire blood feeding period.

The quantitative determination of the overall hemoglobinolytic activity indicates that digestion is almost negligible for the first two days after tick attachment on the host and dramatically increases between days four and six, progressing towards the end of the slow feeding period (Figure 1). The maximum level of hemoglobinolytic capacity (activity normalized to the entire tick gut) was recorded in the guts of fully fed ticks that just dropped off the host. The trend of an increase in total hemoglobinolysis matches with the concomitant activity profiles of the majority digestive enzymes, namely IrCB, IrCC, IrAE and IrCD. The significant up-regulation of their respective mRNA levels could be monitored already at the second day of feeding, peaking towards the end of the slow feeding period between days four and six and then steeply declining during the rapid engorgement phase. This result is consistent with an earlier histological observation demonstrating that RNA is mainly accumulated in the stem and prodigest cells (herein referred to as reserve and initial digestive cells, respectively), that disappear during the rapid engorgement stage [15]. The contrast of the significant down-regulation of mRNA expression to the peaking hemoglobinolysis and enzyme activities (except for IrCL) during the rapid engorgement suggests that the following second continuous digestive phase, i.e. blood digestion in fully fed ticks detached from the host, relies mainly on the enzymes synthesized and accumulated during the slow feeding period. Our results contradict the prevailing opinion that the rapid engorgement stage is associated with reduced hemoglobinolysis, which has been accordingly designated the reduced-digestion phase in the literature [18,20,21]. To our knowledge, the general recognition of the term 'reduced-digestion phase' originates from a 1973 report on the total proteolytic activity in gut homogenates of the hard tick *Hyalomma excavatum *[22]. The authors reported a low activity in both the initial stage of feeding as well as in fully engorged females. However, it is important to take into consideration that they related the proteolytic activity to the total protein concentration in the gut extracts without any compensation for the proteins of the host origin. To our experience, it is impossible to free mechanically the dissected gut tissues of significant host blood contamination, since substantial portion of the host proteins is already present inside the digestive vacuoles. Moreover, a gentle washing has to be applied to prevent an undesired detachment of digestive cells from the epithelium. Therefore, the host proteins inevitably remain the most abundant components in the gut homogenates from the later feeding stages. Despite its simplicity, normalization of hemoglobinolytic and enzyme activities to one tick gut is more reliable than relation to the total protein or host hemoglobin concentrations (based on absorbance at 415 nm) and reflects better the real total proteolytic potential of the gut. In conclusion, this approach leads to a novel interpretation of the blood digestion capacity during the 'big-sip' which disproves the earlier opinions.

The only enzyme activity that drops significantly during the fast engorgement phase is that of IrCL. This finding was also supported by the Western blot analysis showing a substantial reduction of IrCL amount in fully engorged ticks (unpublished results). The reason for the decrease of IrCL activity in fully fed ticks remains still obscure. One possible explanation to this effect is a simultaneous increase of cathepsin L-specific inhibitors, such as cystatins found to be expressed in the midgut of the soft tick *Ornithodoros moubata *[23] or the hard tick *Haemaphysalis longicornis *[24]. Our results strongly imply that *de novo *protein synthesis of the digestive enzymes rather than activation of existing pro-enzymes underlies the overall increase of hemoglobinolytic activity in the gut of *I. ricinus*. The main evidence for this interpretation was provided by Western blot analysis of IrCB from gut homogenates dissected during the course of blood feeding (Figure 4A). At all time points, IrCB was mainly detected as an activated mature enzyme and the proportion of the IrCB pro-enzyme and mature active enzyme did not seem to be altered significantly in time (Figure 4A). The affinity purified antibodies made it possible to perform a clear immunolocalization of IrCB in *I. ricinus *midgut sections prepared at different time points of feeding (Figure 4B). Similar results were obtained for IrCL and IrCD (data not shown), indicating that the entire digestive apparatus remains localized intracellularly in the digestive cells during the feeding. This is in accordance with our previous single time point localization of IrAE in the midgut of semi-engorged *I. ricinus *[25] and digestive enzymes localized in the digestive cells of other tick species [24,26,27]. On the other hand, it remains to be rigorously investigated whether or not intracellular digestion is preserved also in the guts of fully engorged ticks during the second digestive phase, following tick detachment and preceding oviposition.

Based on the results given herein we can conclude that all the major constituents of the tick digestive pathway are regulated at the transcriptional level. Certainly, the mechanisms triggering and regulating the expression of digestive enzymes remain to be explored. The negligible gene expression and enzyme activity at the early stage of tick feeding make the digestive system extremely vulnerable target for efficient impairment, for example via antibodies presents in vaccinated animals. Therefore, digestive enzymes as well as yet unknown regulating factors are promising candidates as concealed antigens for the development of efficient 'anti-tick' vaccines capable to control ticks and associated transmission of tick-borne pathogens [28,29].

## Conclusions

Ticks are obligate blood-feeders capable of ingesting a blood-meal exceeding more than one-hundred times their own body weight. Efficient interference of the blood feeding and digestion processes may be a key to tick control and transmission of tick-borne pathogens. Despite its importance, our knowledge of the molecular mechanisms involved in blood digestion has been rather limited and dispersed across various ixodid tick species. In order to obtain a comprehensive view of blood digestion in a single tick species, *Ixodes ricinus*, we combined genetic and biochemical analyses to unravel the complex suite of gut-associated cysteine and aspartic peptidases [8]. We have demonstrated that the key components of the tick digestive apparatus, namely the cathepsins D, B, L, C and the asparaginyl endopeptidase, are remarkably similar to those found in phylogenetically distant parasites such as nematodes [11], platyhelminthes [9] and even protozoa such as malaria Plasmodium [30]. Moreover, we have deconvoluted how *I. ricinus *gut-specific peptidases function to mediate the gradual hydrolysis of hemoglobin [12]. The present study accomplishes our focus on this multi-enzyme network, as it provides a complex dynamic view of gene expression and enzyme concentration and activities during the course of tick feeding on the host. We have demonstrated that expression and protein synthesis of the component peptidases increase most dramatically towards the end of the slow-feeding period. Overall hemoglobinolysis as well as activities of most peptidases (except cathepsin L) reach their maxima during the rapid engorgement phase, which contradicts the established opinion that blood digestion is reduced during this phase. On the other hand, hemoglobinolysis as well as the activities of individual peptidases are negligible during the first two days after attachment to the host. This feature makes the digestive peptidases, and yet unknown molecules involved in their regulation, promising targets for tick control possibly leading to the rational development of efficient 'anti-tick' vaccines. The applied aspect of this work will be the subject of future reports.

## Methods

### Ticks, feeding and tissue dissection

*Ixodes ricinus *ticks were collected by flagging in localities around Ceske Budejovice, Czech Republic. One hundred *I. ricinus *females were allowed to feed naturally on four laboratory guinea pigs (25 females each) in the presence of the same number of males. At a specific time of feeding, namely 2, 4, 5 and 6 days post-attachment (p.a.), groups of 15 to 20 females were forcibly removed from each guinea pig in a proportional manner using forceps. The last group was allowed to accomplish feeding and drop off the host (7 to 8 days p.a.). Gut tissue from individual time points, including 25 of unfed females (day 0) were dissected under a binocular microscope taking care not to disrupt the epithelium. Gut tissue was subsequently washed from the excess host blood in phosphate buffered saline (PBS). Cleaned gut tissues were subsequently longitudinally divided into two halves and pooled for either RNA isolation or tissue extraction. A smaller number (3 - 4) of dissected tick gut tissues was processed independently for microscopy observations (see below). Laboratory animals were treated in accordance with the Animal Protection Law of the Czech Republic no. 246/1992 Sb, ethics approval number 137/2008.

### Isolation of total RNA and analysis of peptidases expression by quantitative real-time polymerase chain reaction

For isolation of total RNA, the NucleoSpin^® ^RNA II kit (Macherey-Nagel) was used. Pooled gut tissue was placed into RA1 buffer, homogenized with a plastic pestle and further processed according to the manufacturer's instructions. Integrity of the resulted total RNA was analysed using agarose gel electrophoresis according to [31] and stored at -80°C until use. Single stranded cDNA was reversibly transcribed from 0.5 μg of total RNA using the Transcriptor High Fidelity cDNA Synthesis Kit (Roche). Resulting cDNA was diluted 20 times and 2 μl were used as a template for semi-quantitative real-time polymerase chain reaction (qRT-PCR) performed in a final volume of 25 μl using Dual labeled UPL probes (Roche) and specific primers designed online http://www.universalprobelibrary.com (Table 2). The reaction was carried out using the Rotor-Gene RG3000 PCR cycler (Corbett Research) and the amplification conditions were 95°C - 10 min followed by 40 cycles of 95°C - 15 sec and 60°C - 60 sec. All reactions were carried out in triplicates. Data were analyzed and quantified with the Rotor-Gene6 analysis software. Relative values were standardized to the gene for elongation factor 1α (ELF1A) [32] and normalized to the sample with the highest level of expression.

**Table 2:** Primers, probes and conditions used for semi-quantitative real-time PCR

Product	Forward primer 5'-3'	Reverse primer 5'-3'	Annealing Temp. (°C)	**Probe No.**^**a**^	Amplicon size (bp)
**ELF1A**	acgaggctctgacggaag	cacgacgcaactccttcac	60	165	81

**IrCL**	aaccacctggggtgatga	caagaggtatgctagcactgga	60	15	89

**IrCD**	gacagaaggcggacagtacc	cggaaattgtgaaggtgacat	60	78	74

**IrAE**	cgaaaccgtgctttcctg	tcagtcttctcagcgtcacc	60	22	77

**IrCB**	tcaacaagatcaacacaacttgg	tcatggagatggatttgtcg	60	4	60

**IrCC**	caccaagaacagggtgaagaa	ctcgcaaccctgagagtagg	60	15	76

^a ^For the Roche Universal ProbeLibrary Nos. see: https://www.roche-applied-science.com/sis/rtpcr/upl/index.jsp

### Gut tissue extraction

The second half of pooled gut tissues were dispersed in 300 μl of ice-cold 0.1 M sodium acetate buffer pH 5.0 in a 1.5 ml Eppendorf tube and homogenized by three times repetition of freezing in liquid nitrogen, thawing and grinding with a plastic pestle. An aliquot of the homogenate was boiled with reducing SDS-PAGE buffer for Western blot analysis [33]. The remaining homogenate was supplemented with CHAPS to a final concentration of 1%, incubated on ice for 30 minutes under agitation and then cleared by centrifugation (20,000× g, 4°C, 10 min). The supernatant aliquots (referred to as gut tissue extracts, GE) were stored at -80°C until use for activity measurements and active-site titration.

### Determination of the overall hemoglobinolysis and individual peptidase activities

Overall hemoglobinolytic activity in gut tissue extracts was determined using the hemoglobinolytic fluorescamine assay as described previously [12]. The hydrolytic activity of IrCB, IrCC, IrAE, IrCL and IrCD was measured with the Infinite 200 M (Tecan) microplate fluorimeter using fluorogenic substrates and the shielding inhibitors presented in the Table 1. The activity measurements were performed at 25°C in 0.1 M sodium citrate-phosphate (SCP) including 2.5 mM DTT (for cysteine peptidases) and 25 mM NaCl (for IrCC) at the respective pH optimum (Table 1). Gut extracts were appropriately diluted in the assay buffers to achieve roughly 1,500 RFU/min. Enzyme activity was measured for at least 10 minutes and calculated from the linear portion of the kinetic curve. All measurements were performed in triplicates. The measured activity was normalized per one tick gut using the formula: RFU/min/gut = (measured RFU/min × dilution factor)/(number of tick guts × 0.5).

### Determination of the peptidase molar concentrations by active-site titration

The absolute molarity of individual peptidases in the gut tissue extract was determined by the stoichiometric titration method according to Barrett and Kirschke [34] and Knight and Barret [35]. The following inhibitors were applied as active-site titrants: CA-074 for IrCB, Ala-Hph-VS-Ph for cathepsin C, Aza-N-11a for IrAE and pepstatin for IrCD (Table 1), as described previously [12]. An aliquot of the GE was incubated with various amounts of the titrant for 30 min at 35°C in above mentioned SCP buffers at the respective pH optima (Table 1). Residual peptidase activities were measured using the assay systems as described in the previous paragraph, and plotted into the titration curves.

### Expression of recombinant IrCB and preparation of antibody for Western blot analysis and indirect immunofluorescence

To recombinantly express IrCB proenzyme (GenBank ABO26563), the *E. coli *bacterial expression system and Champion™ pET Directional TOPO^® ^Expression kit (Invitrogen) was used. The N-terminal (His)_6_-tagged fusion of IrCB was prepared using the pET100/D-TOPO^® ^expression vector and the following primers: forward, 5'-CACCCGTGAGATTCATCC-3'; reverse, 5'-AGCTATTCGTCCTTGGGG-3'. The resulting expression constructs were transformed into TOP10 cells (Invitrogen) and sequenced using the T7 forward and T7 reverse sequencing primers. The correct constructs were transformed into BL21 Star™ (DE3) *E. coli*. Expression was induced with 1 mM IPTG in LB medium. Isolated inclusion bodies were dissolved in a buffer containing 6 M guanidium hydrochloride and the fusion (His)_6_-tagged IrCB was purified using Ni^2+^-chelating chromatography in the presence of 8 M urea. The purified protein was renatured by dialysis against 0.4 M L-arginine, 0.15 M NaCl, 1 mM mercapthoethanol and gradually decreasing the concentration of urea (from 8 to 0 M) with final dialysis against 25 mM Tris-HCl pH 7.5. Rabbits were immunized with purified soluble recombinant IrCB (recIrCB) using a standard immunization protocol [36]. The immunoglobulin fraction was enriched by serum precipitation with caprylic acid [37] and the resulting antibody (about 0.5 mg protein/ml) referred to as Ra×IrCB_Ig _were used for Western blot analysis following reducing SDS-PAGE [33]. For the purpose of indirect immunofluorescence, the IrCB-specific antibodies were further purified by affinity chromatography using a column of 1 mg recIrCB coupled to 1.5 ml of CNBr-activated Sepharose™ 4B (GE-Healthcare) according to the protocol provided by the manufacturer. Sixty milliliters of Ra×IrCB_Ig _diluted in PBS to 0.15 mg/ml were loaded onto the column, washed with 50 ml of PBS and eluted with 0.2 M L-glycine, 0.15 M NaCl, pH 2.2. The fractions within the eluted peak were immediately neutralized with 1 M Tris-base and pooled. The aliquots of the resulting purified antibodies (150 μg protein/ml) referred to as Ra×IrCB_ACP _(for affinity chromatography purified) were stored at -20°C until use.

### Indirect immunofluorescent localization of IrCB in gut tissues by microscopy

Dissected tissue was fixed overnight in a solution of 4% formaldehyde and 0.1% glutaraldehyde, in 0.1 M sodium phosphate buffer pH 7.3 at 4°C. Samples were subsequently agitated three times for 10 min in washing buffer (4% glucose in 0.1 M sodium phosphate buffer, pH 7.3). Microwave incubations in a water bath were carried out three times for 30 s each in a microwave oven set at the lowest power (80W). Samples were subsequently dehydrated under the microwave radiation using ascending ethanol dilutions, then infiltrated in LR White resin (London Resin Company, Ltd.) in ratios of resin : 95% ethanol 2:1, 1:1, 1:2 (1.5 h each, 4°C) and finally kept in pure LR White resin overnight at 4°C. Samples were then transferred to gelatin capsules (Polysciences, Inc.) and filled with resin, which was allowed to polymerize for 24 h at 50°C. Semi-thin sections (0.5 μm) were prepared, transferred onto glass slides and blocked with 1% BSA and 10% low fat dry milk in PBS-Tween (0.3% Tween-20) for 10 min. Incubation with the primary Ra×IrCBACP antibodies (30 μg/ml) in PBS was performed in a humid chamber for 3.5 h at room temperature. For negative control experiments, the primary antibody incubation was omitted. Sections were washed with PBS-Tween (four times 5 min) and then incubated in a Alexa Fluor^® ^488 dye-conjugated goat anti-rabbit antibody (Invitrogen/Molecular Probes) diluted to 1:500 in PBS-Tween for 1 h at room temperature. After washing with PBS-Tween, the slides were counterstained with DAPI (4',6'-diamidino-2-phenylindole; 2.5 μg/ml; Sigma). Finally, sections were mounted in 2.5% DABCO (1,4-diazabicyclo[2.2.2]octane; Sigma) dissolved in glycerol and examined using the Olympus FW1000 confocal microscope and consequently processed with the Fluoview (FV10-ASW, Version 1.7) software.

## Abbreviations

GE: gut tissue extract; IrCB: *Ixodes ricinus *cathepsin B; IrCL: *I. ricinus *cathepsin L; IrCC: *I. ricinus *cathepsin C; IrCD: *I. ricinus *cathepsin D; IrAE: *I. ricinus *asparaginyl endopeptidase (legumain); p.a.: post-attachment; qRT-PCR: semi-quantitative real-time polymerase chain reaction; RFU: relative fluorescence units.

## Competing interests

The authors declare that they have no competing interests.

## Authors' contributions

ZF, PK, MM and DS designed the study and PK and MM were responsible for its coordination. ZF and DS expressed, purified and refolded the recombinant IrCB and prepared the antibodies. ZF performed and analyzed mRNA expression profiles by qRT-PCR. HF performed tissue dissections, microscope observations, and immunolocalization of IrCB. JK and PK measured and evaluated the enzyme activities and performed the Western blot analysis. MH determined the enzyme concentration by active-site titration and analyzed the data. PK and ZF wrote the paper with the critical input of MM, DS and MH. All authors have read and approved the final version of this manuscript.
